# Diagnostic and Therapeutic Strategy for Vagal Schwannoma: Case Series and Literature Review

**DOI:** 10.3390/medicina59061013

**Published:** 2023-05-24

**Authors:** Antonella Loperfido, Alessandra Celebrini, Bruno Fionda, Gianluca Bellocchi, Giovanni Cristalli

**Affiliations:** 1 Otolaryngology Unit, San Camillo Forlanini Hospital, 00152 Rome, Italy; 2 U.O.C. Radioterapia Oncologica, Fondazione Policlinico Universitario Agostino Gemelli IRCCS, 00168 Rome, Italy; 3 Otolaryngology Unit, Bambino Gesù Children’s Hospital IRCCS, 00165 Rome, Italy

**Keywords:** schwannoma, vagal schwannoma, vagus nerve, extracranial, parapharyngeal space, cervical

## Abstract

*Background and Objectives:* Clinical management of vagal schwannoma is a real diagnostic and therapeutic challenge because the medical history and clinical examination are often non-specific and vagal nerve injury following surgical resection still represents an unsolved problem. The aim of this paper is to provide a case series along with a diagnostic and therapeutic algorithm for vagal schwannoma of the head and neck, combining our experience with clinical evidence available in the literature. *Materials and Methods:* We retrospectively analyzed a series of patients affected by vagal schwannoma who were treated between 2000 and 2020. In addition, a review of the literature on vagal schwannoma management was conducted. Based on the cases described and the literature review, we made a diagnostic and therapeutic algorithm for the management of vagal schwannoma. *Results:* We were able to identify 10 patients affected by vagal schwannoma and treated between 2000 and 2020. All patients presented with a painless, mobile, slow-growing lateral neck mass with onset varying from a few months to years. The preoperative diagnostic workup included ultrasound (US) in nine cases, computed tomography (CT) with contrast in six patients and magnetic resonance imaging (MRI) of the neck in seven cases. All patients included in this study were surgically treated. *Conclusions:* Vagal schwannoma management represents a true challenge for clinicians and surgery is currently the most effective therapeutic strategy. A multidisciplinary approach through the collaboration of otolaryngologist with other specialists is desirable to develop a tailored treatment plan for the patient.

## 1. Introduction

A schwannoma is an uncommon and benign encapsulated nerve sheath tumor with a slow growing attitude that originates from Schwann cells [1]. This pathological condition was first described in 1908 by Josay Verocay, a pathologist from Prague [2].

It is also called neuroschwannoma, neurilemmoma or neurinoma of Verocay [3].

A schwannoma may occur along any cranial, peripheral or autonomic nerve in the body, except for the olfactory and optic nerves as these lack Schwann cells [4]. It has been reported that about 25–45% of schwannomas are found in the extracranial head and neck region [5] and they mainly occur in the lateral part of neck [6]. Neck schwannomas are grouped as medial or lateral. Medials are those arising from the cervical sympathetic chain or the last four cranial nerves (number IX, glossopharyngeal; number X, the vagus; number XI, the accessory and number XII, the hypoglossal). Lateral schwannomas are those arising from the cervical or brachial plexus trunk [7].

Parapharyngeal space is the most common place of the head and neck region where extracranial schwannomas arise, and they are usually of vagal origin [8].

A vagal nerve schwannoma affects both genders equally without a sex related predisposition [9]. It affects any age group, but most cases are found between the third and fifth decades [10]. Malignant transformation from a benign schwannoma is rare; however, it has been described [11].

Pre-operative investigations include ultrasonography (US), computed tomography (CT), magnetic resonance imaging (MRI) and fine-needle cytology (FNAC) [12].

A vagal schwannoma is usually asymptomatic. However, sometimes hoarseness of the voice, due to paralysis of the vocal cords, may be present. Patients can also show a paroxysmal cough on palpating the mass, which is strongly suggestive of vagal nerve origin. In advanced cases when the tumor is large, further symptoms, such as dysphagia, dysphonia, pharyngeal or airway obstruction with related dyspnea, may develop and are suggested to be correlated with schwannoma size [13].

Because of its rarity and lack of neurological deficit as a presenting symptom, preoperative consideration of schwannomas is very difficult. Several differential diagnoses may be entertained for neck tumors, including metastatic cervical lymphadenopathy, submandibular salivary gland tumors, inflammatory cervical lymphadenopathy, carotid body tumors, neurofibroma, lipomas, thyroid cysts or nodules, teratoma or brachial cysts [14].

Even though complete surgical resection is the mainstay of treatment, clinical management of vagal schwannomas is a real diagnostic and therapeutic challenge because not only are the medical history and clinical examination often non-specific, but also vagal nerve injury following surgical resection still represents an unsolved problem [15].

The aim of this paper is to provide a case series along with a diagnostic and therapeutic algorithm for vagal schwannomas of the head and neck, combining our experience with clinical evidence available in the literature.

## 2. Materials and Methods

We retrospectively analyzed a series of patients affected by vagal schwannoma and treated between 2000 and 2020. The inclusion criteria were >18 years old, histologic confirmation of vagal schwannoma and surgery as treatment of choice. The exclusion criteria included patients without histological confirmation of vagal schwannoma, patients referred for follow-up or for radiotherapy. Clinical history, preoperative evaluations, surgical data, macroscopic and histological appearance of the lesions and postoperative morbidity were obtained. All patients were treated according to the principles of good clinical practice and gave their approval for publication of the cases. Data from the selected ten cases were collated and processed using the Data Analysis ToolPak loaded in Excel to calculate descriptive statistics.

In addition, a review of the literature on vagal schwannoma management was conducted. Two independent authors screened citations in titles and abstracts, present in the main medical databases, including PubMed, Scopus and the Cochrane Library, in order to identify appropriate papers. Eligible citations were retrieved for full-text review. The time period considered included the published articles available within the databases from their inception until March 2023. The search strategy included a combination of the following terms: “cervical”, “vagal”, “nerve” and “schwannoma”. Based on the cases described and the literature review, we made a diagnostic and therapeutic algorithm for the management of vagal schwannoma.

## 3. Results

We were able to identify ten patients affected by vagal schwannoma and treated between 2000 and 2020; five were male and five were female (F:M = 1:1). The mean age was 52.7 years with a range from 23 to 67 years.

One patient was affected by neurofibromatosis type 2 (NF2).

All patients presented with a painless, mobile, slow-growing lateral neck mass with the onset varying from a few months to years.

The preoperative diagnostic workup included ultrasound (US) in nine cases, computed tomography (CT) with contrast in six patients and magnetic resonance imaging (MRI) of the neck in seven cases. In most cases, the patient underwent US followed by CT and/or MRI.

**Figure 1:**
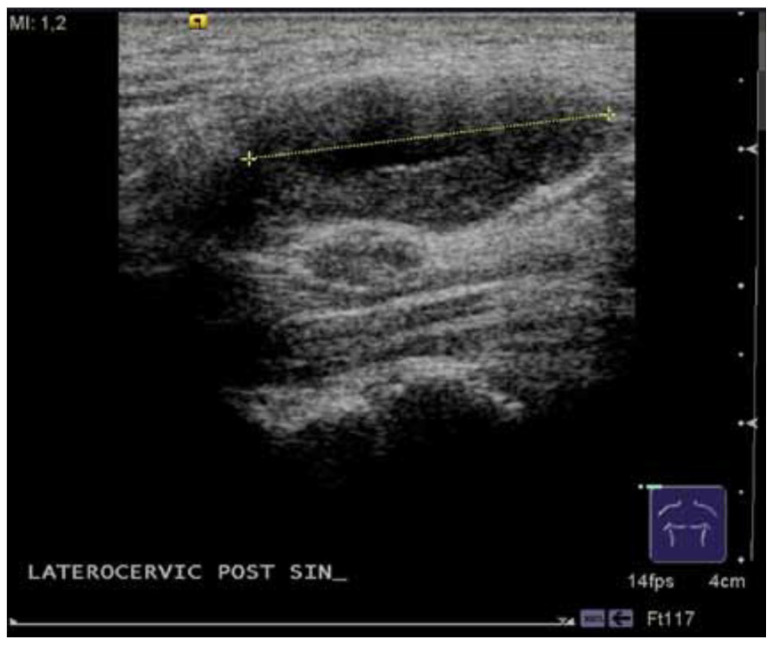
Ultrasonography of a vagal schwannoma.

The images of the US typically showed a well-defined, ovoid or round mass with regular margins and mixed content due to the presence of a central fluid area. The ultrasound appearance included different findings: we found homogeneous content in four patients, calcifications in two cases and fluid components in three cases (Figure 1).

Most of these tumors appeared hypodense or isodense when compared with adjoining skeletal muscles, with variable enhancement after administration of the contrast medium.

**Figure 2:**
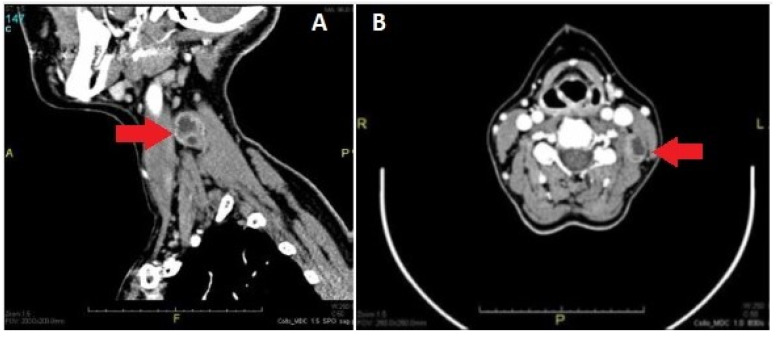
CT imaging of a vagal schwannoma (red arrow) on the left cervical side: (**A**) sagittal view; (**B**) axial view.

Figure 2 shows the CT image of a vagal schwannoma on the left cervical side presenting as a neck mass on the left side, medial to the sternocleidomastoid muscle, with polycyclic margins, partially fluid content and homogeneous enhancement in the peripheral component.

**Figure 3:**
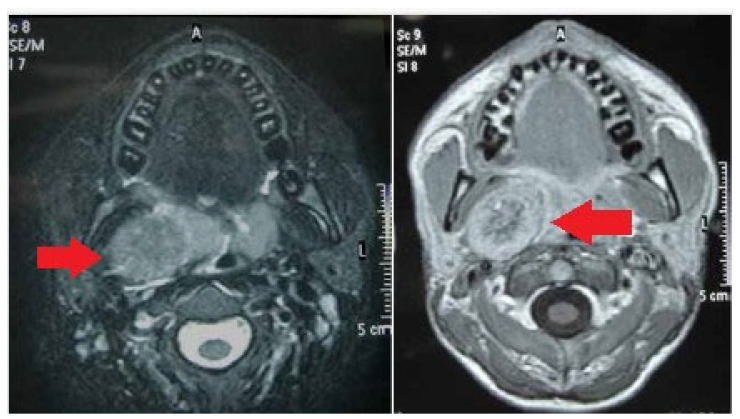
MRI imaging of a vagal schwannoma (red arrow) on the right cervical side.

At MRI, all schwannomas revealed relatively low signal intensity on T1-weighted imaging and signal hyperintensity on T2-weighted imaging. Figure 3 reports a case of vagal schwannoma on the right cervical side, shown on MRI presenting as a homogeneous lesion in the parapharyngeal space on the right side, weakly hyperintense at T2 relatively compared with adjoining skeletal muscles. After contrast administration, the enhancement appeared heterogeneous and predominantly peripheral.

**Figure 4:**
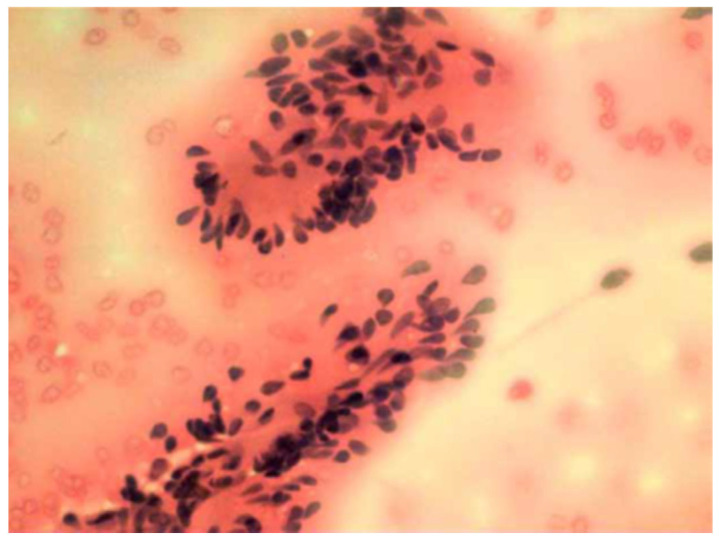
Cytological examination of vagal schwannoma: aggregates of cells with oval and elongated nuclei with finely dispersed chromatin.

In addition, six patients performed fine needle aspiration cytology (FNAC) of the lesion; however, it was not diagnostic in two cases. In three cases, the exam reported the benignity of the lesion. In one case, the cytology was able to diagnose the nature of schwannoma (Figure 4).

All patients included in this study were surgically treated. Extracapsular resection was the treatment of choice for seven patients, while three patients underwent intracapsular excision. The full cohort was approached through a transcervical incision. In none of the histological specimens were we able to identify any malignant transformation.

**Figure 5:**
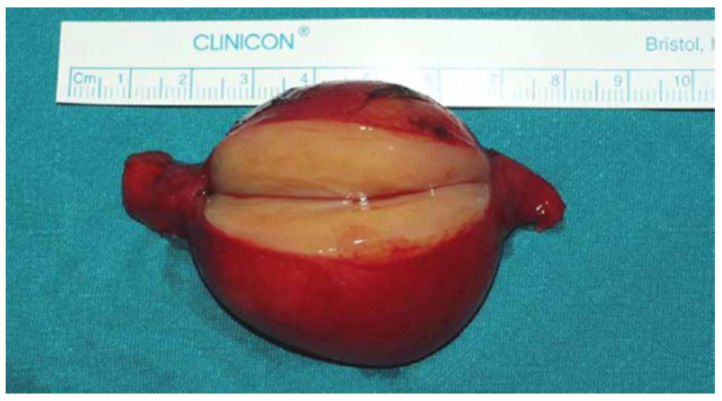
Macroscopic examination of vagal schwannoma.

As reported in Figure 5, on macroscopic examination schwannomas typically appeared as an oval, well-circumscribed and encapsulated neoplasm with a smooth surface and a yellowish white color when cut. The origin of nerve termination was clearly evident.

**Figure 6:**
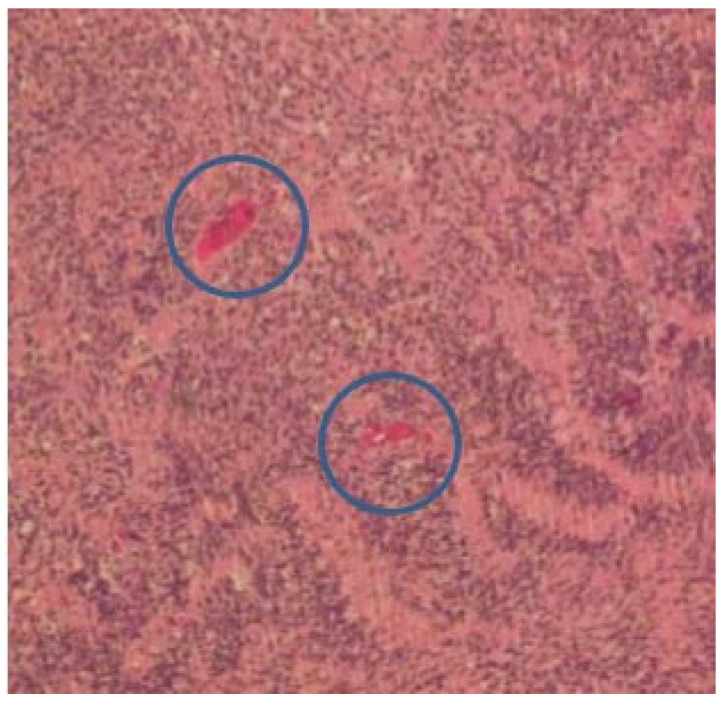
Histopathology of vagal schwannoma: spindle cells organized in small fascicles. The blue circle highlights the Verocay bodies.

The pathological examination of these cases confirmed the diagnosis of a benign schwannoma of the vagus nerve. The mean tumor size was 3.6 cm with a range of 1 to 5 cm. Figure 6 demonstrates the typical histological aspect of the lesion. Microscopically, the neoplasm is composed of spindle cells organized in small fascicles. Verocay bodies with spindle cells, organized in a palisading fashion, can be observed.

Six patients reported postoperative sequelae: the predominant postoperative symptom was hoarseness, observed in all six patients, both associated and not with other less-frequent symptoms such as Horner’s syndrome, found specifically in two patients, or increased heart rate, reported in one case.

Vocal cord palsy was directly confirmed with a laryngoscopy in four patients; one of them had a complete recovery.

A summary of the clinical features of the patients included in this study is shown in Table 1.

## 4. Discussion

A schwannoma is a benign tumor originating from cranial, peripheral and autonomic perineural Schwann cells. It represents 5% of all benign soft tissue tumors and can rarely undergo malignant degeneration [16].

A schwannoma is considered a slow-growing neoplasm. De Araujo et al. and Zhang et al. reported annual tumor growth rates of 3 mm and 2.75 mm, respectively [17,18].

Approximately 25–45% of these tumors occur in the head and neck region, most frequently involving the parapharyngeal space. In this anatomical district, the vagus nerve represents the most common nerve of origin [19].

In our series, the patients were men and women in equal prevalence with a mean age of 52.7 years. Langner et al. and Lahoti et al. also reported that the disease affects both sexes equally, with a higher incidence between the third and fifth decades of life [20,21].

One of our patients was affected by NF2, an autosomal dominant disease characterized by the presence of multiple tumors involving the central nervous system. In particular, a vestibular schwannoma represents the most common intracranial tumor associated with NF2, which is typically bilateral in these patients [22].

Other authors describe cases of vagal schwannomas in the context of NF2; in particular, bilateral forms are more generally found in association with this syndrome [23].

Vagal schwannomas are considered as a true challenge for clinicians. First, it is difficult to obtain a specific preoperative diagnosis because the signs and symptoms are commonly not specific. In the early stage of the disease, the most common symptom is a solitary, slow-growing mass in the neck that can be palpated along the medial border of the sternocleidomastoid muscle. There are also cases of schwannomas found incidentally in asymptomatic patients [1].

In our series, apart from the cervical mass, no patients presented with additional onset symptoms, such as hoarseness, coughing, dysphagia, dyspnoea, headache or vagal syndrome.

In later stages of the tumor, patients can show signs and symptoms that depend on tumor size, location and nerve of origin and they may include hoarseness, dysphagia, dyspnoea, Horner’s syndrome, coughing, painless cheek swelling or other neurological deficits [24].

The most common symptom of a vagal schwannoma is hoarseness, due to vocal cord palsy. Occasionally, a paroxysmal cough may be produced on palpation of the mass. This is due to vagal stimulation, representing the pathognomonic sign for vagal schwannomas [25].

Therefore, a paroxysmal cough during palpation of a mass located on the medial border of the sternocleidomastoid muscle should make the clinician suspect a vagal schwannoma.

In treatment planning for a schwannoma, it is fundamental to determine the origin of the tumor to preserve the nerve function. Schwannomas of the vagus nerve displace the internal jugular vein laterally and carotid artery medially; schwannomas from the cervical sympathetic chain displace the carotid artery and jugular vein without separating them [12].

Clinically, schwannomas and neurofibromas are often misdiagnosed as both being benign peripheral nerve sheath tumors. The main difference is that schwannomas are eccentric to the nerve of origin, whereas neurofibromas are spindle-shaped and located in the center of the perineum [26].

A preoperative approach with imaging modalities is essential for a correct diagnosis, providing data about the location, size and extent of the tumor that represent basic aspects to define the surgical operation. CT and MRI are also helpful to define the nerve of origin with the aim of reducing postoperative neural deficits [27].

In general, the diagnostic investigations reported in the literature are ultrasonography (US), computed tomography (CT), magnetic resonance imaging (MRI) and fine needle aspiration cytology (FNAC) [28]. However, schwannomas are frequently difficult to characterize on FNAC, and for some authors FNAC has not been helpful in the diagnosis [1,29].

The usefulness of FNAC is controversial because its diagnostic accuracy depends greatly on the quality of the specimen and the experience of the cytopathologist. In addition, most authors do not recommend open biopsy or FNAC for these tumors because they may increase the risk of bleeding, hematoma and hoarseness [30,31].

Ultrasonography may play a role in the diagnosis of vagal schwannomas, especially in those rare cases where the diameter of the nerve of origin is large and the tumor is connected to a clearly delineated nerve [32].

US images of schwannomas are typically defined by a round or elliptical cross-section with a clear edge and an internal echo reflective of histology. Patterns may be homogenous-to-heterogeneous and cystic changes may be found [33].

On CT, schwannomas usually have an inhomogeneous appearance with a well-defined mass associated with internal cysts and peripheral enhancement [24].

MRI is the most sensitive and specific preoperative approach, allowing for a more accurate identification of the nerve of origin.

On MRI, schwannomas are characterized by specific signs (split fat, fascicular, target) and signal patterns (T1-weighted images show low signal intensity, and T2-weighted images display high intensity) [19]. Some authors have demonstrated a schwannoma as a lesion with a specific peripheral hyperintense rim and central low intensity on enhanced T1 images [34]. Moreover, MRI may better define the relationship between the schwannoma and its nerve of origin than CT.

At the pathological examination, the schwannoma is an encapsulated lesion representing a solid or cystic spindle cell mesenchymal tumor. The diagnosis can be confirmed by biopsy using electron microscopy and immunohistochemical analysis, in which schwannomas are usually positive for S-100 protein and Leu-7. Common histological features include the presence of fibrous capsule, Antoni A and Antoni B areas, cellular zones and Verocay bodies. Antoni A and Antoni B areas represent typical architectural histological features of schwannomas [35,36]. Type A tissue is highly cellular and demonstrates nuclear palisading and associated Verocay bodies, which reflects their prominent extracellular matrix and secretion of laminin. Type B tissue is characterized by hypocellularity with large myxoid tissue. A typical Verocay body, considered diagnostic for schwannoma, is constituted by horizontal rows of palisaded nuclei alternating with acellular zones consisting of cytoplasmic Schwann cell processes [37].

Ancient schwannomas are a subtype of schwannoma pathologically typified by degenerative changes, vascular sclerosis, calcification, ossification cystic necrosis, relative loss of Antoni type A tissue and degenerative nuclei that may be misinterpreted as sarcomatous pleomorphisms [38].

Differential diagnoses of vagal schwannoma include neurofibroma, paraganglioma, metastatic nodes, schwannoma of the cervical sympathetic chain, lymphomas, glomus vagal tumors/carotid body tumors, branchial cleft anomalies, thyroid cysts, parathyroid cysts and vascular malformations of the neck. Some authors claim that blood samples and sometimes even genetic testing could be necessary for diagnosis. It is of paramount relevance to make a distinction between vagal nerve schwannoma and the other possible differential diagnoses. For example, if on radiologic imaging a schwannoma could not be distinguished from neck paraganglioma, FNAC is not recommended, and it is even dangerous to perform due to the risk of hypertensive crisis after FNAC [39].

Paraganglioma typically shows early arterial enhancement on CT; it represents a hypervascular lesion (whereas a nerve sheath tumor is hypovascular) and it is usually characterized by the typical aspect of MRI-defined “salt and pepper”, due to scattered flow voids [40]. Metastatic lymph nodes are often multiple and only the case of a single metastasis from an unknown primary could become a possible differential diagnosis with a schwannoma [41]. The differential diagnosis from a schwannoma of the cervical sympathetic chain can be done by paying attention to the pattern of splaying the carotid bifurcation, as they behave differently [42].

When it comes to dealing with neurofibromas of the head and neck region, they are much rarer than schwannomas. Researchers tend to believe that the most likely precursor of neurofibromas—that is to say, the perineural fibroblast—is a more primitive neuroectodermal cell than the Schwann cell from an embryological point of view. Such tumors intertwine themselves within several fascicles of origin. Removing neurofibromas is, therefore, more difficult than resection of schwannomas and is more likely to result in functional loss. Unlike schwannomas, neurofibromas are not associated with the finding of Antoni A and B areas. When analyzing them with the microscope, they are composed of interlacing fascicles of wavy elongated cells that usually contain abundant collagen. Myxoid and degenerative areas are also not as frequent in neurofibromas as in schwannomas. Neurofibromas may be classified as being localized, plexiform or diffuse; however, more than 90% are of the localized variety [38].

In the treatment of extracranial head and neck schwannomas, surgical indications should be based carefully on the balance between benefits and the risk of nerve palsy after excision.

As for the management of schwannomas, different treatment approaches have been proposed such as waiting and seeing, radical tumor excision or intracapsular enucleation [43].

Typically, the choice of approach depends on the size of the tumor, its location and its relation to adjacent vessels.

The surgical approach is the treatment of choice, but the slow growth and the non-invasive nature of this lesion justify the observational approach as well. The main surgical options include radical excision with nerve grafting, intracapsular enucleation and debulking [44].

Radical excision involves the total removal of the tumor while sacrificing the nerves of the perineum connected to the tumor. Therefore, nerve grafting is planned.

Intracapsular resection is a more conservative treatment which involves the preservation of the tumor capsule linked to the perineum and the removal of only the central part of the tumor. Tumor debulking is associated with a higher risk of recurrence. Even with intracapsular dissection, it could be difficult to preserve nerve integrity, so, preoperatively, patients should be well informed about the possibilities of neurologic deficits. The main goal of surgery should always be tumor excision with the least possible neurologic deficit. Whichever technique is employed, the nerve of origin is likely to be affected. The rate of nerve palsy following complete excision and intracapsular dissection is reported to be 100% and 67%, respectively [45].

The preferred method to remove the tumor is intracapsular enucleation because the complications are usually transient and, in most cases, do not require treatment; in fact, some authors report a neural function preservation of more than 30%, compared with radical tumor resection with a primary anastomosis [46].

Interestingly, several studies have shown that understanding the eccentric growth pattern of the tumor and the anatomical features of the recurrent laryngeal and non-laryngeal reflexes can help surgeons to protect nerves as much as possible to preserve good vocal function after surgery [47,48].

In addition, some authors propose as a strategy for functional preservation in the treatment of extracranial schwannomas of the head and neck: the use of an electromyography (EMG) system during tumor resection could help to prevent motor nerve paralysis. A similar kind of strategy has been implemented for clinical use in thyroid and parotid gland surgery [49].

It is widely reported in the literature that if the nerve cannot be preserved during surgery, primary repair or a nerve graft should be performed using a microsurgical technique, with or without medialization of the vocal cord [39].

Finally, some authors suggested a wait-and-see approach, postponing surgery only in case of the symptoms’ worsening, with neural weakness becoming clinically relevant [50].

Common postoperative complications following the removal of a vagal nerve schwannoma are due to the origin of the tumor directly from the nerve fibers. They include nerve injury, vocal cord palsy and hoarseness of voice. Therefore, preoperative speech and swallowing evaluation, as well as post-operative management, are critically important in voice and swallowing rehabilitation in these patients.

Specifically, hoarseness is reported by most patients following schwannoma resection, whereas vocal cord paralysis occurs in 85% of patients after tumor resection. Further common complications of schwannoma resection include pharyngolaryngeal anesthesia, aspiration and cranial nerve IX, XI and XII palsies, which may be transient or permanent [51]. Finally, other uncommon complications described include Horner’s syndrome and alteration of heart rate [52].

In our case series, we report two cases of Horner’s syndrome as post-surgical sequelae. This syndrome is characterized by ipsilateral palpebral ptosis, pupillary miosis and facial anhidrosis. It is a rare complication of neck surgery that can result from direct injury to the cervical sympathetic chain during tumor excision or indirect injury due to traction on the sympathetic chain. Indirect injuries generally recover spontaneously over time with the resolution of Horner’s syndrome [53].

We also report a case of a patient with the onset of heart rate alteration after surgery. It is widely documented in the literature that the vagus nerve slows the heart rate [54].

Those patients unsuitable for surgery may benefit from an observational approach (“wait and see”) or, if symptomatic, from radiotherapy, although there is not enough strong evidence in the literature for that approach. In fact, there is limited evidence on the efficacy of radiotherapy for schwannomas of the head and neck compared to the acoustic nerve, which is largely treated with radiotherapy [55].

There is, however, emerging evidence encouraging the use of radiotherapy in schwannomas of other cranial nerves (III, IV, V and VI) in non-surgical candidates, resulting in the local control rate ranging from 90 to 100% [56,57].

Specifically with regard to the radiobiological behavior of vagal schwannomas, some authors have underlined that one of the main limitations to the efficacy of radiotherapy relies on the intrinsic radioresistance of this entity [21].

For this very reason, in similar clinical conditions the typical schedule choice reported in the literature is typically hypofractionated and implies the use of stereotactic radiotherapy [58].

Some preliminary experiences have shed light on the possible role of proton beam radiotherapy which, using heavy particles, may overcome the intrinsic radioresistance of vagal schwannomas and allow for extremely high local control and favorable nerve functional protections rates [59].

When considering radiotherapy as an alternative therapeutic approach in surgical candidates, however, it is necessary to take into account the occurrence of acute adverse events; such adverse events are usually self-limiting and may improve by administering low doses of steroids. Dumbbell-shaped and large-volume tumors are significant predictive factors for the occurrence of such acute side effects [60].

With regard to the possibility of following a wait-and-see strategy, it is fundamental to propose such an approach only to patients who are willing and compliant to undertake regular visits and imaging examinations as prescribed by the physician; in fact, protocol could be switched to active treatment in cases of radiologic growth, pain or new cranial nerve dysfunction [61].

In order to tailor the optimal therapeutic strategy, a multidisciplinary management is desirable [62].

**Figure 7:**
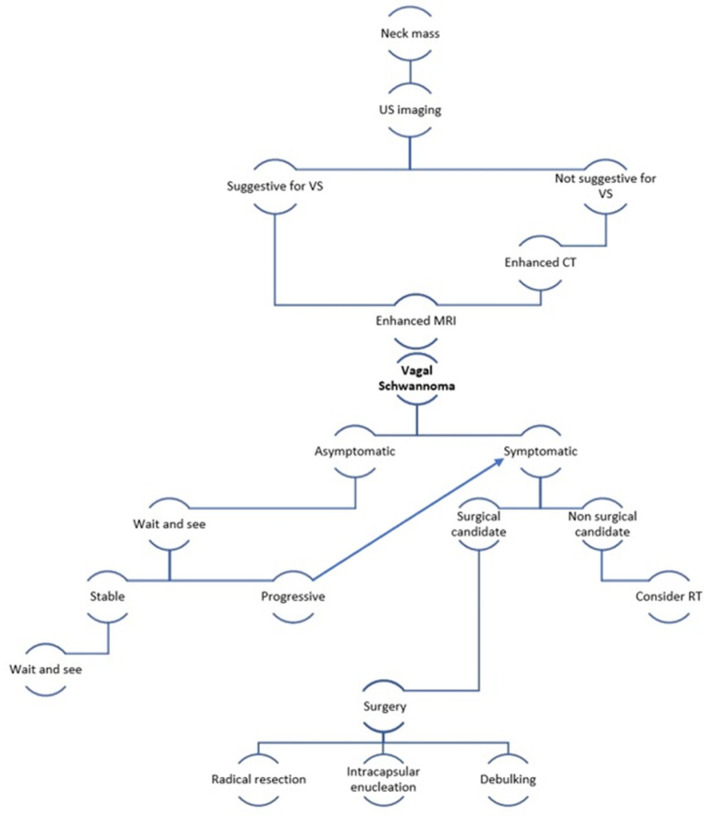
VS = vagal schwannoma; non-surgical candidate = frail patient after geriatric assessment or inoperable due to comorbidities or refusing surgery; RT = radiotherapy.

In Figure 7, we propose an overview with a diagnostic and therapeutic algorithm for the management of vagal schwannomas of the head and neck.

## 5. Conclusions

The clinical management of vagal schwannomas is definitively challenging both in terms of diagnosis and treatment choice. In fact, symptoms are usually non-specific, therefore resulting in possible diagnostic delays. In addition, even though complete surgical resection is the mainstream therapeutic strategy, it should be carefully considered that post-operative complications, such as vagal nerve injury, still represent major problems. We have provided a management algorithm on vagal schwannomas, combining our clinical experience with scientific evidence available in the literature.

## Figures and Tables

**Table 1:** Patients’ Features.

Feature	Result
Number of patients	10
M/F	5/5
Mean Age	52.7 ys (23–67)
Average tumor size	3.6 cm (1–5)
US	9
FNAC	6
CT	6
MRI	7
Treatment approach	Surgery
Post-surgical sequelae	Hoarseness: 6
	Recurrent nerve palsy: 4
	Horner’s syndrome: 2
	Alteration of heart rate: 1

## Data Availability

Not applicable.
